# Genetics and Molecular Biology of Head and Neck Cancer

**DOI:** 10.3390/biom11091293

**Published:** 2021-08-31

**Authors:** Krzysztof Szyfter

**Affiliations:** Institute of Human Genetics, Polish Academy of Sciences, 60-479 Poznan, Poland; krzysztof.szyfter@igcz.poznan.pl

Head and neck cancer (HNC) is a multistep process proceeding from single gene mutations generated by carcinogens to the substantial dysregulation of metabolic processes. Tobacco smoking, a primary casual factor in HNC, provides a number of carcinogens. Recent years a large effort has been made to obtain insight into the mutational signature of cancer cells [1]. To some extent two papers of this Special Issue deal with unraveling of this mutational profile. Borkowska et al. [2] searched for a *PIK3CA* gene mutation in HNSCC. It was established that the standard RT-qPCR is not sensitive enough as compared with droplet digital PCR. The down estimation of the *PIK3CA* gene mutation may be followed by an incorrect therapeutic decision. The topic of the paper by Ustaszewski et al. [3] is a comparison of microarray gene expression profile. The authors recommended to use cultured epithelial cells as a control instead of material taken from a surgical margin.

The molecular biology of HNS is still insufficiently recognized, and several points require more findings to create a better understanding. A study on the role of RNA methylation performed on cell lines has shown a positive correlation between selected RNA methylation machinery and methylated N6-adenosine abundance. It was concluded that specific mRNA methylation may influence tumorigenesis [4]. The aim of the next paper by Kowalski et al. [5] is to estimate an interaction of programmed death receptor 1 (PD-1) with its ligand involved in the regulation of the immune system. It was found that the greater is tumor size, the expression of two studied molecules is higher. The authors suggest testing the applicability of anti-PD1 and anti-PD-L1 antibodies to laryngeal cancer therapy [5].

Another two articles are involved search for biomarkers of HNC progression. Lubiński et al. [6] attempted to evaluate a risk of survival of laryngeal cancer subjects in relation to zinc level. Low levels of zinc concentration tend to increase the risk of death. Next, a review paper by Liu et al. [7] hypothesizes that desmosomes acting in the cell as intercellular adhesion complexes when abnormally expressed deregulate the proliferation, invasion, migration, morphogenesis and apoptosis of cancer cells. However, their role in tumor suppression still remains unclear.

Another three papers deal with micro-RNA’s involvement in HNC tumorigenesis. Coon and Kingsley [8], studying the expression of mir-365, established a correlation of its expression with metalloproteinases that may in turn contribute to increased cell growth. Janiszewska et al. [9] established a direct interaction of mir-1290 with MAF transcription factor acting as suppressor of laryngeal cancer. The role and usefulness of micro-RNA as a biomarker of HNC is presented in review paper by Kabziński et al. [10].

One can believe that at least some gaps in general knowledge concerning genetics and molecular biology of head and cancer were covered by the content of the presented articles.
